# Ultrasound-Guided Percutaneous Release Procedures in the Lumbar Ligamentum Flavum by Acupotomy: A Cadaveric study

**DOI:** 10.1155/2019/2807901

**Published:** 2019-11-23

**Authors:** Xinyue Zhu, Yifeng Shen, Zixiang Liu, Peiliang Gu, Shiliang Li, Weiguang Zhang

**Affiliations:** ^1^Department of Acupuncture and Moxibustion, China-Japan Friendship Hospital, No. 2 East Yinghua Road, Beijing 100029, China; ^2^Beijing First Hospital of Integrated Chinese and Western Medicine, Department of Rehabilitation Medicine, No. 13, Jintai Road, Chaoyang District, Beijing 100029, China; ^3^Hospital of Chengdu University of Chinese Medicine, Sichuan, Chengdu 610072, China; ^4^Health Science Center, Peking University, No. 38 Xueyuan Road, Beijing 100191, China

## Abstract

**Objective:**

This study aims to determine the methods of percutaneous release procedures in the lumbar ligamentum flavum (LF) under ultrasound guidance by acupotomy and provide an anatomical basis for intrusive treatment of lumbar disc herniation and lumbar spinal canal stenosis.

**Methods:**

Twelve cadavers including 4 females and 8 males aged 60 to 90 years (73.42 ± 14.57 years), without formalin fixation, were selected. Guided by an ultrasound transducer, we punctured acupotomy to release lumbar LF in L3/L4, L4/L5, and L5/S1 segments. In the transverse-axis approach, the probe was placed transversely, while in the longitudinal-axis approach, the probe was placed longitudinally. The depth of needle penetration (A), the distance between the puncture point and spinous process (B), and the distance between the puncture point and sacral cornu (C) were measured on cadavers, and the depth of needle penetration (U-A), the distance between the puncture point and spinous process (U-B), and the angle for acupotomy (D) on ultrasound images were also measured. Statistical analyses were carried out using SPSS. Paired sample *t*-tests and homogeneity of variance tests and one-way analysis of variance (ANOVA) were performed. The Pearson correlation coefficients and linear correlation coefficients were calculated for the data obtained from ultrasound and cadaver measurements.

**Results:**

No obvious blood vessels and nerves were observed in the puncture path, and the spinal dura was intact. There was no statistical difference between the left and right side measurements obtained from the ultrasound images and the cadavers. The penetration depth in the transverse-axis approach was less than that in the longitudinal-axis approach, and the angle of the needle in the transverse-axis approach was greater than that in the longitudinal-axis approach. The measured data for the transverse-axis approach for L3/L4, L4/L5, and L5/S1 segments showed that there were no differences in the needle angle, the depth of needle penetration, and the distance from the spinous process to the puncture point among the three segments. There was a strong correlation between the depth of needle penetration and the distance from the spinous process to the puncture point on the ultrasonic images and the cadavers on the path of acupotomy. Linear equation A = 2.02 + 0.83 *∗* U-A, *R*^2^ = 0.352; B = 1.37 + 0.71 *∗* U-B, *R*^2^ = 0.252, where A/B refers to the data measured on the cadavers and U-A/U-B refers to the data measured on the ultrasound images.

**Conclusion:**

In this study, ultrasound guidance was applied, which better guaranteed the safety and feasibility of acupotomy therapy. Before performing the treatment, the depth of needle penetration in the human body can be determined by measuring the distance between the needle point and the target position on the ultrasound image. Under ultrasound guidance, the transverse-axis approach has a smaller puncture depth and greater puncture angle than the longitudinal-axis approach. Hence, this study believes that the transverse-axis approach is safer for the clinical application of ultrasound-guided LF acupotomy lysis.

## 1. Introduction

Middle-aged and elderly individuals with lumbar disc herniation (LDH) and lumbar spinal canal stenosis (LSCS) often have hypertrophy of the lumbar ligamentum flavum (LF). Lumbar LF hypertrophy can directly compress the spinal canal, which in turn compresses the cauda equina of the corresponding segment and leads to lower limb symptoms. In addition, as the LF is involved in forming a part of the posterior margin of the intervertebral foramen [1, 2], it determines the anteroposterior diameter of the intervertebral foramen, intervertebral disc, and other soft tissues posterior to the lumbar vertebrae [3]. With the protrusion of the lumbar intervertebral disc, the LF hypertrophies due to abnormal mechanical stress [1, 2]. The joint action of the two, in turn, reduces the anteroposterior diameter of the intervertebral foramen, thereby leading to symptoms of nerve root compression.

However, the treatment of patients with LDH, LSCS, and LF hypertrophy is still unclear. Most patients with pain only receive treatment for the herniated disc, whereas the stimulation of the spinal canal and nerve roots by LF hypertrophy is often ignored. This could fail to alleviate the symptoms of some patients after treatment. In clinical practice, acupotomy has achieved significant short- and long-term clinical efficacy in the treatment of patients with lumbar disc disease caused by LF hypertrophy [4–7]. Acupotomy can cut and detach the abnormal, cicatricial, and contractured tissues by causing only microtrauma. Acupotomy has been widely used clinically by doctors practicing traditional Chinese Medicine, orthopedics, and pain department in China with a satisfactory efficacy. Acupotomy is mainly used to treat chronic injuries of the motor system, cervical and lumbar diseases, and degenerative diseases of bones and joints, such as tenosynovitis, muscle injury, periarthritis of shoulder, cervical spondylosis, lumbar disc herniation, knee osteoarthritis, heel pain, etc. However, traditional acupotomy only determines the puncture site and depth based on surface anatomical landmarks of the human body and the physician's tactile feedback from the acupotome. These types of operations are not performed under direct vision and present certain operational difficulties and risks.

In this study, ultrasound guidance was combined with dissection to develop a specific approach for the acupotomy lysis of the lumbar LF. We hope that this will provide a more accurate method of ultrasound-guided acupotomy lysis in clinical practice, as well as a safe and reliable treatment for patients with LDH, LSCS, and LF hypertrophy.

## 2. Materials and Methods

### 2.1. General

**Figure 1:**
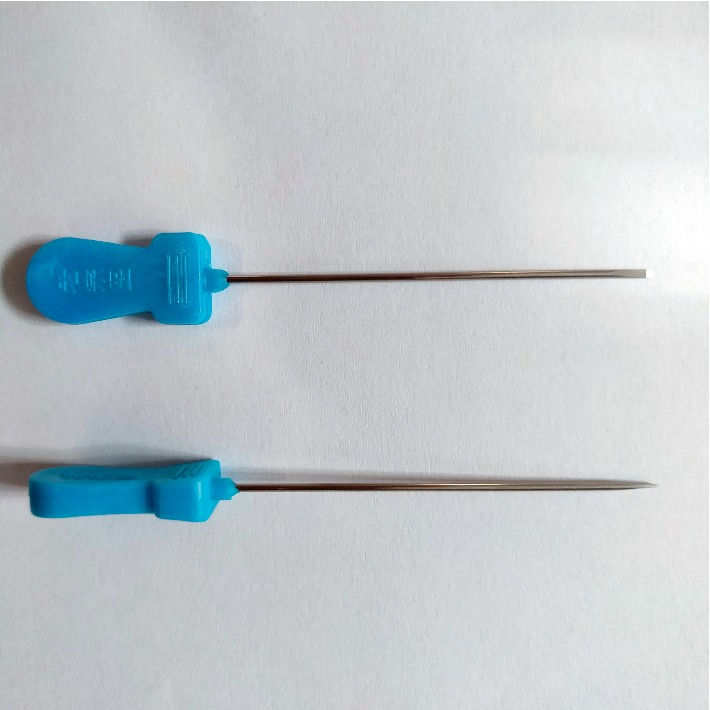
Front and side view of the acupotomy.

Twelve fresh cadavers (4 women, 8 men) without formalin fixation, aged 60–90 years (73.42 ± 14.57), were selected. None of the cadavers had lumbar injury; cadavers with deformities, trauma, and clear degeneration were excluded. An ultrasound system (Wisonic Medical Technology Co., Ltd., model: Wisonic-Navi) and convex array ultrasound probe (2–5 MHz) were used. Water acupotome (length: 90 mm, diameter: 1.0 mm) was used in the study. An acupotome is a miniature surgery instrument consisting of a handle, needle body, and blade (Figure 1). The water needle knife is designed on the basis of needle knife which can inject liquid from pipe inside the needle.

### 2.2. Ultrasound-Guided Acupotomy Lysis Technique

The cadavers were placed in a prone position, and the midline was marked. The convex array probe was used to scan upwards from the sacrum along the longitudinal axis, to identify and mark the fifth lumbar spinous process (L5), fourth lumbar spinous process (L4), and third lumbar spinous process (L3). Acupotomy lysis of the LF was conducted under ultrasound guidance using two different approaches at three segments: L3/L4, L4/L5, and L5/S1.

**Figure 2:**
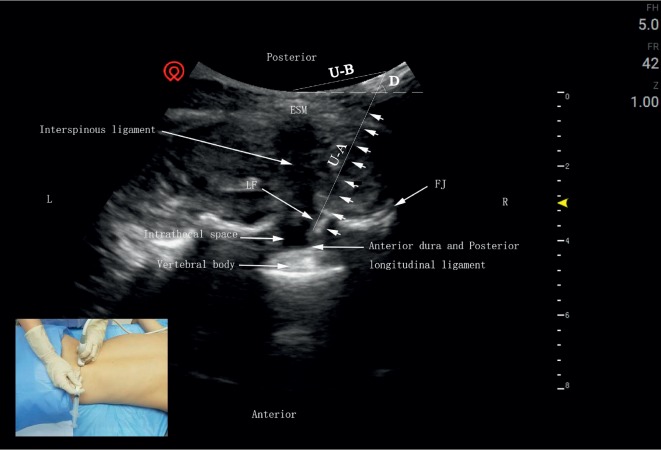
Transverse-axis approach: puncture needle of the median coronal sonogram of the lumbar spine. The intrathecal space is a hypoechoic area between the hyperechoic ligamentum flavum and the anterior dura and posterior. The short arrows refer to the needle knife position. ESM, erector spinae muscle; ES, epidural space; LF, ligamentum flavum; FJ, facet joint; L, left; R, right; U-A, the depth of acupotome penetration; U-B, the distance from the puncture site to the spinous process; D, the angle of acupotomy.

Transverse-axis approach (Figure 2): the probe was first placed on the marked L4 spinous process in the direction of the longitudinal axis and moved slightly downward. When the midline of the L4 and L5 spinous processes was confirmed, the probe was quickly rotated 90° and placed transversely. The puncture point is close to the ultrasonic probe, and the needle is inserted in the plane to obtain the shortest puncture path. The acupotome was inserted along the lateral plane of the probe, and the direction of puncture was constantly adjusted according to the ultrasound image and the tactile feedback from the acupotome. Saline bolus was administered as the needle was inserted, and changes in resistance were detected at each level until finally reaching the LF. Obvious resistance could be detected at this point via the saline injection resistance test [8]. The acupotome was retained, and measurement data were recorded.

**Figure 3:**
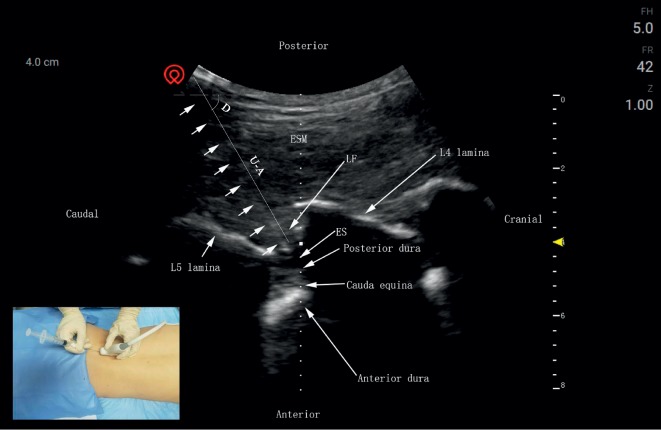
Longitudinal-axis approach: puncture needle of the paramedian sagittal sonogram of the lumbar spine. The posterior epidural space is a hypoechoic area between the hyperechoic ligamentum flavum and the posterior dura. The short arrows refer to the needle knife position. ESM, erector spinae muscle; ES, epidural space; LF, ligamentum flavum; U-A, the depth of acupotome penetration; D, the angle of acupotomy.

Longitudinal-axis approach (Figure 3): the probe was placed longitudinally, slightly toward the facet joint between the spinous processes. The puncture point is close to the ultrasonic probe, and the needle is inserted in the plane to obtain the shortest puncture path. The acupotome was inserted laterally along the probe while keeping the acupotome within the plane of the ultrasound image until it finally reached the LF. Obvious resistance could be detected using the saline injection resistance test. The acupotome was retained, and measurement data were recorded.

### 2.3. Local Anatomy of Lumbar Acupotome Retention Site

**Figure 4:**
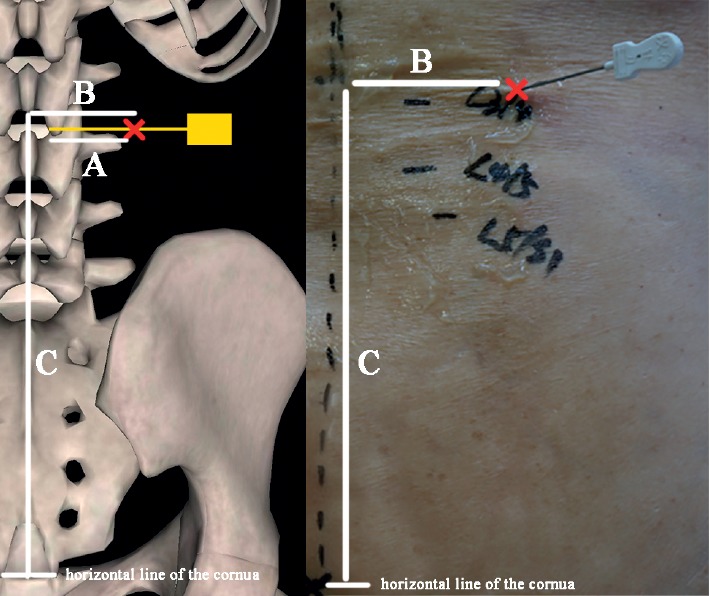
Measuring in the cadavers. A, the depth of acupotome penetration; B, the distance from the puncture site to the spinous process; C, the vertical distance from the puncture site to the horizontal line of the cornua sacralia; red Cross, puncture site; yellow, acupotomy.

The depth of acupotome penetration (A), the distance from the puncture site to the spinous process (B), and the vertical distance from the puncture site to the horizontal line of the cornua sacralia (C) were measured from the cadavers (Figure 4). The depth of acupotome penetration (U-A), the distance from the puncture site to the spinous process (U-B), and the angle of acupotomy (D) were measured from the ultrasound image.

The skin along the midline of the lumbar spinous process was dissected till the acupotome retention site, and the soft tissue was separated layer-by-layer. The structures, blood vessels, and nerves along the puncture path were observed, and each layer was photographed. The position of acupotome retention was observed after the LF was exposed. Finally, the LF was resected to expose the spinal canal anterior to the lamina of the vertebral arch to observe the integrity of the dura mater.

### 2.4. Statistical Analysis

All data were analyzed using the statistical software, SPSS 20.0. Paired sample *t*-tests were performed between the left and right sides of the cadavers and between the two approaches for A, B, U-A, U-B, C, and D. Paired sample *t-*tests were performed between the two approaches for A, B, U-A, U-B, C, and D. Homogeneity of variance tests and one-way analysis of variance (ANOVA) were performed to compare the A, B, U-A, U-B, and D between the transverse- and longitudinal-axis approaches for the three segments. Measurement data were all expressed as mean ± standard deviation ($\bar{x}\pm s$). The Pearson correlation coefficients and linear correlation coefficients were calculated for the data obtained from ultrasound and cadaver measurements. The linear equation for the ultrasound-measured U-A and U-B with the cadaver-measured A and B were performed.

## 3. Results

### 3.1. Left and Right Side Ultrasound versus Cadaver Measurements

In the L3/L4, L4/L5, and L5/S1 segments, there were no statistically significant differences (*P* > 0.05) between the left and right sides of the ultrasound and cadaver measurements in the transverse and longitudinal-axis approaches. This indicates that there were no differences in the measurements between the left and right sides for the same acupotomy approach in the same segment (Table 1).

### 3.2. Comparison of Ultrasound-Guided Acupotomy between the Transverse-Axis Approach and Longitudinal-Axis Approach

Acupotomy lysis of the lumbar LF was performed at L3/L4, L4/L5, and L5/S1 via two approaches. The specific data on acupotomy distance, depth, and angle are shown below (Table 2). The values for A, U-A, C, and D were compared between the transverse-axis approach and longitudinal-axis approach. The results indicated that there were significant differences between the two approaches for A, B, and U-A (*P* < 0.01). Comparison of the mean values indicates that the A and U-A of the transverse-axis approach were smaller than those of the longitudinal-axis approach, whereas B was larger than that of the longitudinal-axis approach. Comparison of D between the two approaches showed that the differences were not significant for the L4/5 and L5/S1 segments (*P* > 0.05) but were significant for the L3/L4 segment (*P* < 0.05). This suggests that the puncture angle of the transverse-axis approach was larger than that of the longitudinal-axis approach. The results indicate that the puncture depth of the transverse-axis approach was smaller than that of the longitudinal-axis approach, whereas the puncture angle was larger. Having a shallower puncture depth is safer in clinical practice. Furthermore, the rules of ultrasound imaging suggest that a larger the puncture angle would give clearer images. Thus, the results indicate that the transverse-axis approach is superior to the longitudinal-axis approach.

### 3.3. Comparison of the L3/L4, L4/L5, and L5/S1 Segments Using the Transverse-Axis Approach

U-A, U-B, A, B, and D were compared among the three segments under the transverse-axis approach. There were no significant differences in the homogeneity of variance (Levene's test statistics were 1.671, 0.323, 1.747, 0.061, and 0.111, respectively; the *P* values were 0.196, 0.725, 0.941, 0.182, and 0.895, respectively, all >0.05). The differences in U-A, U-B, A, B, and D among the three segments were not statistically significant (*F* test statistics were 0.246, 0.907, 2.054, 0.458, and 1.212, respectively; and the *P* values were 0.782, 0.409, 0.634, 0.137, and 0.304, respectively, all >0.05).

The same steps were repeated to compare the U-A, A, B, and D among the three segments under the longitudinal-axis approach. There were no significant differences in the homogeneity of variance (Levene's test statistics were 0.312, 0.685, 1.498 and 0.207, respectively; the *P* values were 0.733, 0.508, 0.232, and 0.813, respectively, all >0.05). The differences in U-A, A, B, and D among the three segments were not statistically significant (*F* test statistics were 0.097, 1.351, 1.231, and 0.127, respectively; and the *P* values were 0.907, 0.267, 0.299, and 0.881, respectively, all >0.05).

### 3.4. Pearson Correlation Coefficients and Linear Equations of Ultrasound and Cadaver Measurements

**Figure 5:**
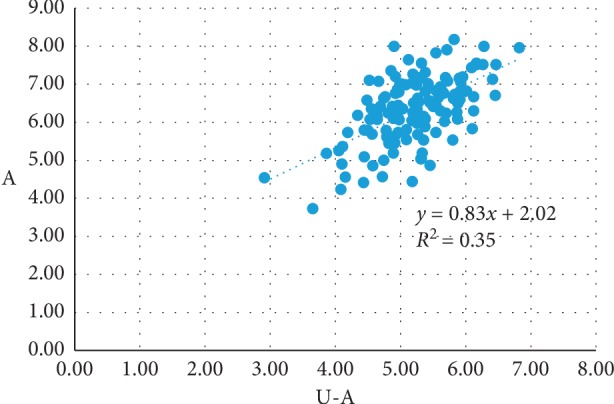
The linear regression diagram of A versus U-A.

**Figure 6:**
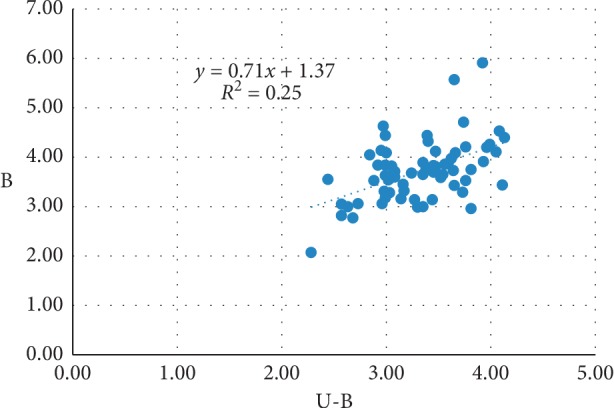
The linear regression diagram of B versus U-B.

Pearson correlation analysis showed that the Pearson correlation coefficient of U-A for all segments (ultrasound measurements) with A (cadaver measurements) was 0.593 (*P*=0.000 < 0.05), and the Pearson correlation coefficient of U-B for all segments (ultrasound measurements) with B (cadaver measurements) was 0.502 (*P*=0.000 < 0.05), thus indicating that U-A and U-B had a significant linear correlation with A and B (Figures 5 and 6). This implies that there is a strong correlation between the experimental data obtained by ultrasound and that from actual measurements. The linear equation for the ultrasound-measured U-A and U-B with the cadaver-measured A and B was cadaver measurements A = 2.02 + 0.83 *∗* U-A, *R*^2^ = 0.352; B = 1.37 + 0.71 *∗* U-B, *R*^2^ = 0.252. This suggests that the depth measured from the ultrasound images can be used to obtain the puncture depth in actual human bodies.

### 3.5. Anatomy of the Lumbar Region

**Figure 7:**
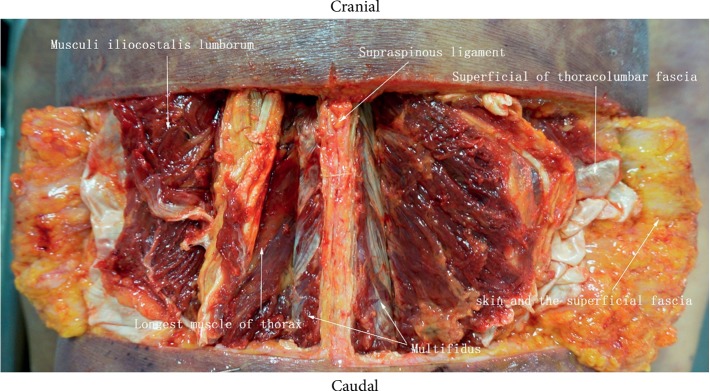
Anatomical sketch map.

**Figure 8:**
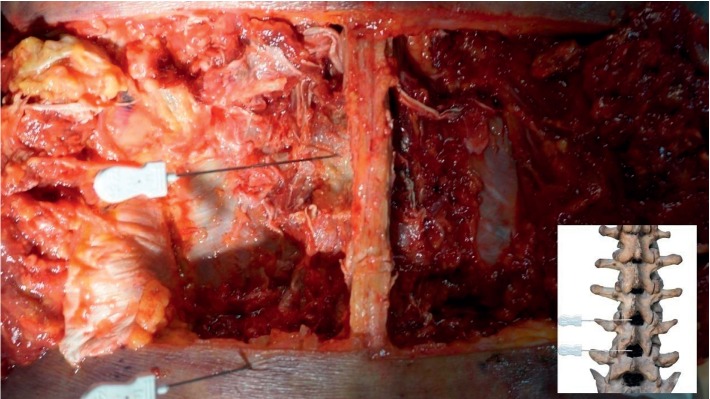
Needling into the yellow ligament.

**Figure 9:**
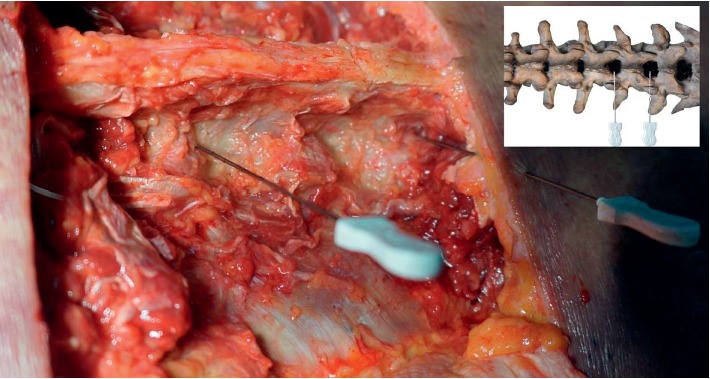
Needling into the yellow ligament from the lateral view.

**Figure 10:**
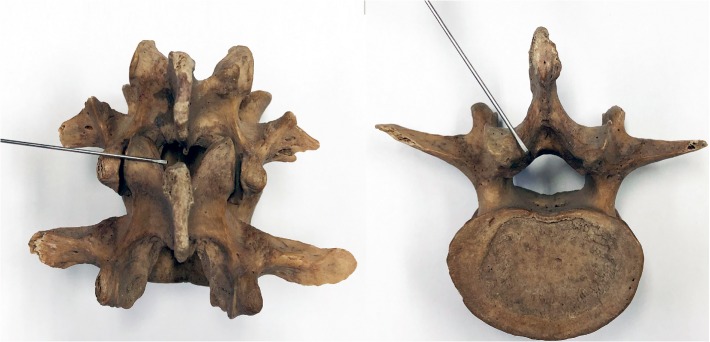
Needling into the yellow ligament inside the facet joint (schematic diagram).

Anatomical observations revealed that the puncture path of the acupotome passed through the skin, superficial thoracolumbar fascia, musculus iliocostalis lumborum, musculus longissimus thoracis, multifidus muscle, and LF (Figure 7). No major blood vessels or nerves were observed in the puncture path. The LF, interspinous ligament, and supraspinous ligament were slightly yellow under direct observation. All three closely adhered between the spinous process and vertebral arch lamina. The site of acupotome retention was at the interlaminar LF on the inside of the facet joint (Figures 8–10). The exposed spinal canal anterior to the vertebral arch lamina and the dura mater was intact. This suggests that the puncture approach for ultrasound-guided acupotomy lysis of the LF can penetrate into the lumbar LF and will not damage major blood vessels, nerves, or dura mater in its puncture path.

## 4. Discussion

**Figure 11:**
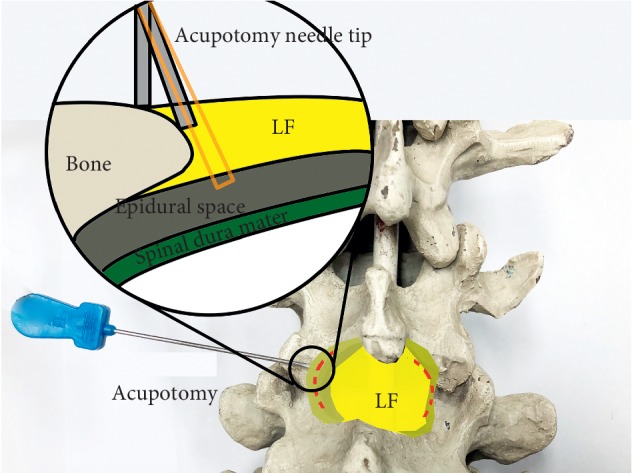
The acupotomy treatment area stripped and peeled 4 or 5 times to form the cut trace of discontinuous line. Needle tip operation on the bone surface without a feeling of falling to avoid puncturing the spinal dura mater. LF, ligamentum flavum. Orange rectangle: acupotomy is not in the bone surface.

Clinical studies [9–12] have shown that LF hypertrophy is one of the main causes for significant lower back and leg symptoms in patients with LSCS. Acupotomy lysis of the LF has currently achieved good clinical efficacy in patients with LDH [4, 6, 13, 14]. The acupotome is a medical device that penetrates the human body with a needle and can achieve various therapeutic effects. It can exert acupuncture effects by stimulating acupuncture points and also serve as a scalpel for cutting, dissecting, and other actions. As the acupotome is inserted into the body like an acupuncture needle, it causes minimal damage during cutting and dissecting [15]. Usually, in acupotomy, the part to be treated must be stripped and peeled 4 or 5 times to form the same cut trace of discontinuous line. Such an operation can form a row of small holes in the thickened yellow ligament and bone attachment to decompress and repair (Figure 11). The diameter of acupuncture needle used in acupotomy is usually 0.4–1.2 mm, similar to the size of a syringe needle. Therefore, acupotomy has the shovel and lysis effect from the insertion and puncture operation in human tissue that the human body can basically repair spontaneously, with an extremely low possibility of scar formation. The difference between the lysis in acupotomy and the complete breaking of the fiber in surgical operation is that the former belongs to a local fiber disconnection and the human body can perform the repair mechanism after the operation. Following LF acupotomy lysis, the abnormal stress caused by LF hypertrophy will be alleviated to a certain extent, which will decompress the posterior of the spinal canal and reduce its compression of the dural sac and nerve root. However, acupotomy is a blind operation that lacks objectivity and safety [15]. If acupotomy lysis for the LF is not performed correctly, the same complications as those of puncture in lumbar anesthesia can occur. Hence, investigating acupotomy treatment using visualization to ensure the safety and effectiveness of acupotomy therapy has become a key issue for future development. Studies have reported the use of CT-guided LF acupotomy lysis in patients with LSCS [16, 17]. However, not only did the studies require the guidance of C-Arm, but the treatment process was complicated. The patients were also exposed to a certain amount of radiation. Hence, this method cannot be promoted for widespread application. In this study, ultrasound guidance was applied, which better guaranteed the safety and feasibility of acupotomy therapy. Ultrasound guidance does not involve exposure to radiation. Compared with the C-Arm, ultrasound can provide real-time images, is noninvasive, safe, convenient, and flexible. It is therefore more suitable for clinical application in acupotomy therapy.

Ultrasound-guided acupotomy has achieved satisfactory results in patients with scapulohumeral periarthritis, knee osteoarthritis, and cervical spondylosis [18–20]. However, to our knowledge, studies on ultrasound-guided LF acupotomy lysis have not yet been reported. This study applied ultrasound guidance to visualize the entire acupotomy operation completely. The tissues targeted by acupotomy could be displayed through ultrasound images. Lumbar examination using a low-frequency ultrasound probe can be used to observe bony structures, such as the lumbar spinous processes, vertebral laminae, facet joints, and transverse processes, and soft tissues such as the erector spinae, interspinous ligament, and LF. In this study, in-plane acupotome insertion was selected for the transverse- and longitudinal-axis approach. This ensures that the positions of the acupotome tip and body observed in real-time during the operation, thus preventing the full penetration through the LF into the spinal canal caused by the inability to determine the position of the acupotome. In addition, ultrasound enables the real-time monitoring of blood flow. Although blood flow could not be monitored in the cadavers used in this study, the blood flow of the target lumbar segment could be monitored by ultrasound during clinical applications to avoid unnecessary injury.

With regard to the selection of the segment for ultrasound-guided LF acupotomy lysis, the thickness of the LF is not uniform across all segments of the human lumbar spine. LF hypertrophy of the L4/L5 segment was the most significant, followed by the L3/L4 and L5/S1 segments, and LF thickness is directly proportionate to age [2, 21]. Furthermore, clinically, degenerative changes in the lumbar spine mostly occur in the L4/L5 and L5/S1 segments. Therefore, this study performed measurements for ultrasound-guided transverse-axis and longitudinal-axis approach at the L3/L4, L4/L5, and L5/S1 segments. This preliminary experiment compares the advantages and disadvantages and safety of the two puncture pathways. Anatomical observations indicated that the transverse- and longitudinal-axis approaches were able to penetrate the LF, and the puncture path did not involve any major blood vessels, nerves, or dura mater. In clinical practice, ultrasound-guided approaches that are convenient and safe must satisfy the following criteria: (1) have a simple and easy method to operate ultrasound guidance, where the images can display stable and reliable anatomical structures and clearly indicate the acupotomy path; (2) shallow depth of acupotomy puncture that is not susceptible to penetration of the spinal canal. This study has shown that the transverse-axis approach has a smaller puncture depth and greater puncture angle than the longitudinal-axis approach. The rules of ultrasound imaging state that a larger puncture angle will give a clearer image. The transverse-axis approach of acupotomy lysis ensures that acupotomy imaging is clear and the puncture depth is small. Furthermore, in the longitudinal-axis approach of LF acupotomy lysis, the acupotome is parallel to the spinal canal, which increases the operational risk due to the easier access to the spinal canal [8]. Hence, this study believes that the transverse-axis approach is safer for the clinical application of ultrasound-guided LF acupotomy lysis.

Previous studies on ultrasound-guided therapies have yet to compare the depth measurements of images obtained by low-frequency ultrasound probe with the actual depth of puncture. In this study, we found that the puncture depth measured in the cadavers were significantly different from that measured in the ultrasound sector images. We consider that this situation is caused by the extrusion of the probe during operation. At the same time, in the fan-forming image of the low-frequency probe, fewer sound beams are observed on both sides of the probe, which causes the difference between the measured value and the actual value measured on ultrasonographic images. Hence, the two groups of data were analyzed, and the results showed that there was a significant linear correlation between the two, which gave a linear equation. In clinical applications, the linear equation can be used preoperatively to calculate the depth from the puncture site to the target position on the ultrasound image, to obtain a reference value for safe puncture depth required during the actual operation. While referring to experimental data during the clinical use of ultrasound-guided LF acupotomy lysis, close attention must also be paid to the real-time changes of ultrasound images and the patient's reactions in conjunction with tactile feedback from the acupotome. There are explicit risks of dural damage and consequent cerebrospinal fluid leakage due to LF thickness variability and the proximity to the dural sac [22] Needle tip operation should be performed on the bone surface, if there is a feeling of falling with the needle, it indicates that the needle tip has broken through the ligamentum flavum and should not be excessively deep to avoid puncturing the spinal dura mater (Figure 11). If the positions of the deep tissues and acupotome tip cannot be displayed during the operation, the acupotome should not be blindly inserted at a greater depth to avoid unnecessary damage. We suggest that further research like the present cadaveric ultrasonographic study is needed to confirm the safety of the procedure before performing it in patients.

## Figures and Tables

**Table 1:** Left and right side measurements on transverse-axis approach and longitudinal-axis approach.

Left and right-side measurements in the same approach and same segment			U-A (mm)	U-B (mm)	A (mm)	B (mm)	C (mm)	D (°)
Transverse-axis approach	L3/L4	Left	48.11 ± 9.92	31.53 ± 4.18	58.17 ± 7.19	34.20 ± 5.22	153.79 ± 23.51	58.92 ± 5.63
		Right	48.84 ± 6.73	33.61 ± 4.03	55.77 ± 10.46	37.00 ± 4.76	154.49 ± 24.54	56.77 ± 4.65
	L4/L5	Left	48.30 ± 5.83	33.18 ± 3.78	60.52 ± 7.19	35.49 ± 3.96	129.68 ± 19.80	56.53 ± 5.57
		Right	51.23 ± 5.70	35.74 ± 6.56	61.51 ± 6.75	39.54 ± 8.22	128.64 ± 18.85	53.24 ± 6.55
	L5/S1	Left	48.96 ± 8.56	33.38 ± 5.33	62.76 ± 4.76	37.43 ± 5.99	107.47 ± 15.55	55.01 ± 5.42
		Right	51.07 ± 5.32	34.02 ± 4.38	57.61 ± 7.75	37.84 ± 7.47	110.51 ± 12.26	53.54 ± 6.73

Longitudinal-axis approach	L3/L4	Left	53.78 ± 5.94	—	64.98 ± 8.20	11.18 ± 11.56	122.33 ± 23.66	54.69 ± 5.41
		Right	53.83 ± 6.59	—	66.61 ± 9.13	11.97 ± 4.1	122.88 ± 22.93	51.62 ± 5.27
	L4/L5	Left	55.36 ± 5.47	—	66.98 ± 6.35	6.93 ± 5.20	99.75 ± 16.56	53.21 ± 5.86
		Right	53.46 ± 5.42	—	67.14 ± 9.89	11.28 ± 2.32	98.71 ± 16.70	51.32 ± 6.34
	L5/S1	Left	53.23 ± 6.46	—	68.55 ± 4.26	8.75 ± 3.58	80.82 ± 12.78	51.14 ± 8.07
		Right	54.78 ± 5.99	—	70.69 ± 7.99	12.00 ± 5.60	84.58 ± 15.09	54.21 ± 3.91

D, angle of acupotomy puncture; C, vertical distance from the puncture site to the horizontal line of the cornua sacralia; B, distance to the spinous process; A, depth of acupotome penetration measured in cadavers; and U-A, depth of acupotome penetration.

**Table 2:** Paired *t*-test measurements between transverse- and longitudinal-axis approaches.

Comparison of the two approaches in the same segment		U-A (mm)	U-B (mm)	A (mm)	B (mm)	C (mm)	D (°)
L3/L4	Transverse axis	48.48 ± 8.30	32.57 ± 4.15	56.97 ± 8.86	35.60 ± 5.09	154.14 ± 23.51	57.85 ± 5.17
	Longitudinal axis	53.80 ± 6.13^a^	—	65.79 ± 8.53^a^	11.58 ± 8.52^a^	122.61 ± 22.79^a^	53.15 ± 5.46^a^

L4/L5	Transverse axis	50.00 ± 5.95	34.46 ± 5.40	61.01 ± 6.84	37.51 ± 6.64	129.16 ± 18.91	54.88 ± 6.18
	Longitudinal axis	54.16 ± 5.50^a^	—	67.06 ± 8.13^a^	9.11 ± 4.52^a^	99.23 ± 16.27^a^	52.27 ± 6.05

L5/S1	Transverse axis	50.01 ± 7.06	33.70 ± 4.78	60.18 ± 6.82	37.63 ± 6.63	108.99 ± 13.78	54.27 ± 6.03
	Longitudinal axis	54.00 ± 6.14^a^	—	69.62 ± 6.35^a^	10.38 ± 4.88^a^	82.70 ± 13.81^a^	52.67 ± 6.40

^a^Statistically significant differences between the transverse-axis approach and longitudinal-axis approach (*P* < 0.05). D, angle of acupotomy puncture; C, vertical distance from the puncture site to the horizontal line of the cornua sacralia; B, distance to the spinous process; A, depth of acupotome penetration measured in cadavers; U-A, depth of acupotome penetration; U-B, the distance from the puncture site to the spinous process.

## Data Availability

The data used to support the findings of this study are available from the corresponding author upon request.
